# Image registration and subtraction in dynamic magnetic resonance lymphangiography (MRL) of the legs

**DOI:** 10.1259/bjrcr.20210237

**Published:** 2022-04-25

**Authors:** Michael Mills, Kristiana Gordon, Lakshmi Ratnam, Malou van Zanten, Peter S Mortimer, Pia Ostergaard, Franklyn A Howe

**Affiliations:** 1 Molecular and Clinical Sciences Research Institute, St George’s University of London, London, UK; 2 Lymphovascular Medicine, Dermatology Department, St George’s Hospital, London, UK; 3 Department of Radiology, St George’s Hospital, London, UK

## Abstract

Dynamic contrast-enhanced magnetic resonance lymphangiography (DCE-MRL) is regularly reported as unable to depict lymphatic vessels in healthy limbs. In this study, we aim to improve lymph vessel conspicuity with appropriate registration and subtraction of a reference baseline image. Five unaffected individuals and a single unilateral primary lymphoedema patient were recruited to undergo fat suppressed 3D *T*_1_ weighted spoiled gradient echo imaging of the lower limbs at 3.0 T. Images were quality assessed by two physicians and a medical physicist following registration via one of six registration pipelines, and subtraction of the first post-contrast dynamic image (PC1). Wilcoxon non-parametric testing was performed to compare image quality ranking *vs* the unregistered images and inter-rater reliability estimated using intraclass correlation coefficient. Signal enhancement curves were also computed in lymphatic vessels for two participants. Subtraction images were considered to improve lymphatic visibility, and three registration pipelines significantly (*p* < 0.05) outranked those without registration. Those registered to PC1 with an affine and elastic approach were rated best quality (*p* = 0.006). Moderate inter-rater reliability was observed (intraclass correlation coefficient = 0.71) and signal enhancement behaviour appears affected by registration when motion is evident across the DCE-MRL series. We therefore conclude that lymphatic vessel visibility in DCE-MRL images can be improved with registration and baseline subtraction.

## Introduction

The lymphatic system is a whole-body vascular network pivotal in tissue fluid homeostasis and immunity. Failure of the lymphatic system can cause an accumulation of interstitial fluid within the tissues (Lymphoedema) and interruption of immune cell trafficking leading to infection. Lymphoedema is estimated to affect at least 400,000 people in the UK and as many as 250 million people worldwide.^1,2^

The use of MRI in lymphoedema diagnosis and staging has garnered recent interest, with both non-contrast fluid-sensitive *T*_2_ weighted and *T*_1_ weighted dynamic contrast-enhanced magnetic resonance lymphangiography (DCE-MRL) capable of demonstrating the lymphatic vasculature in lymphoedema patients. DCE-MRL appears most sensitive to the visualisation of lymphatic vessels, however anatomic assignment of enhancing structures is confounded by concomitant venous enhancement.^3,4^ Visualising lymphatic vessels in unaffected individuals is also commonly reported as problematic with vessels simply not observed, possibly resulting from reduced size and number of vessels compared to dilated and hyperplastic lymphatic vessels in many, but not all, lymphoedema patients.^5^

Dynamic imaging enables observation of the time course of enhancement in the imaged volume and has been shown capable of estimating bulk bolus flow speeds within lymphatic vessels, a potential proxy for lymph flow speed.^6,7^ In one such study,^7^ vessel signal was interrogated following rigid body registration across the dynamic series in order to compensate for participant motion and improve the accuracy of signal enhancement curves. The authors also subtracted the first image collected from all subsequent dynamics in order to study signal changes from baseline. To our knowledge, this is the only research article referencing the use of image registration and baseline subtraction for DCE-MRL image series. Non-lymphatic DCE studies are routinely registered to, and subtracted from, a reference image to improve contrast agent conspicuity and accuracy of quantitative analyses however.^8^ Deformable, or ‘elastic’, image registration techniques are often preferred in breast DCE-MRI^9–11^ and, given that rigid registration was shown to be insufficient in lengthy DCE-MRL studies of the arms,^12^ we hypothesise that these will be preferential in DCE-MRL of the lower limbs also.

Registration of DCE-MRL data requires the choice not only of registration technique (*e.g.* rigid *vs* elastic) and optimisation metric, but also the selection of an appropriate reference image. The DCE-MRI literature details many reference images including the pre-contrast volume, the first post-contrast volume (PC1), and a sequential (referred to here as ‘rolling’) registration, whereby each dynamic image is registered to the preceding dynamic, *e.g.* PC1 is used as the reference for PC2 and this registered volume used for PC3 and so on.^12–14^

In order to better understand lymphatic dysfunction and disease, investigations focussing on the lymphatics in healthy individuals are required such that normal anatomy and physiology can be established. This is particularly true for MRI as explained prior, therefore in this study we assess the effect of image registration and subtraction on DCE-MRL image quality in healthy individuals. We employ affine only or affine and deformable registration pipelines based on three concepts: registration to PC1, as previously employed in arm MRL studies; registration on a rolling basis, under the expectation that motion between adjacent volumes will be small scale and easier to correct; and registration to a computed volume produced by either averaging across the dynamic series (3D-mean intensity projection or MeanIP) or taking the maximum voxel values across the series (3D-maximum intensity projection or MIP), as these volumes will demonstrate vascular enhancement which may not be present in PC1. We also investigate the effect of registration on signal enhancement curves obtained in lymphatic vessels.

## Methods

### Participants

DCE-MRL data from six participants, enrolled in a larger study of lymphatic imaging (approved by the London – Camden & Kings Cross-Research Ethics Committee, 20/LO/0237), were selected for this study. These participants include five healthy individuals (four female, one male, age = 39.1 ± 18.7 years) recruited from St George’s University and a single female (age = 19.1 years) diagnosed with unilateral primary lymphoedema recruited from St George’s Hospital. Written informed consent was obtained from all participants prior to imaging.

### MR imaging

Participants were imaged feet-first supine at 3.0 T (Dual TX Achieva, Phillips Medical Systems, Netherlands) with a 16-element torso receive coil. Lower limbs were imaged with a protocol including fat suppressed (SPAIR, inversion time = 97 ms) 3D *T*_1_ weighted fast spoiled gradient echo (SPGR): TR/TE = 3.7/1.6 ms, flip angle = 12^o^, number of averages = 1, acquired voxel size = 1 × 1 × 1 mm (zero-filled to provide 0.7 × 0.7 × 0.7 mm voxels). A pre-contrast volume was acquired followed by ≥ 30 minutes of dynamic SPGR after contrast administration and repositioning. Dynamic imaging was performed in order to observe the temporal behaviour of contrast enhancement in the limb. Partial Fourier (k space coverage = 0.6) and SENSE (parallel imaging factor = 1.6 in one in-plane and out-of-plane direction) were used to accelerate imaging. Field of view and temporal resolution varied slightly between participants, however imaging from ankle to knee with 3 minute dynamic images were typical. A time delay of between 2 and 11 minutes elapsed from the start of contrast injection in the limbs under investigation and the commencement of DCE-MRL imaging for the healthy volunteers and approximately 5 minutes in the participant with lymphoedema.

### Contrast injection

A mixture of 0.45 ml Dotarem^®^ (Dotarem, Guerbet, [Gd]=0.5 M, molecular radius ~10 Å^15^), 0.1 ml anaesthetic (1% lidocaine) and 0.45 ml saline was injected intradermally in each of the four interdigital spaces of the foot^1^ (4 ml total volume per limb). Imaging re-commenced after repositioning at isocentre: the participant was moved through the bore to expose the feet for contrast administration in the scanner room. Injection was performed by hand at a rate much slower than typical intravenous administration. Our protocol administers a reduced Gadolinium dose compared to the majority of DCE-MRL studies to both lower the risk associated with contrast administration and reduce venous signal.^3,4,7^ No interventions (*e.g.* injection site massage) were performed post-injection.

### Image processing

Prior to any further processing, image data were split in to left and right limb. Only one limb from each participant was carried forward for further analysis, ensuring that this limb had received the contrast agent solution described above.

Image registration was performed using a software package capable of performing both affine and deformable image registration (NiftyReg^16^). NiftyReg employs normalised mutual information (NMI) and a bending energy penalty term (P_BE_) to create a registration objective function:

$O=\alpha \times NMI-\beta \times P_{BE}$

where α and β are weighting terms which sum to 1.^16^

Post-contrast limb data were registered via: Method 1 – affine registration to the first post-contrast dynamic (PC1); Method 2 – deformable registration to PC1 after initial affine transformation; Method 3 – as Method 2 but registered on a rolling basis (*i.e.* affine and elastic registration of PC2 to PC1, affine and elastic registration of PC3 to the transformed PC2 etc.); Method 4 – as Method 2 but registered to the 3D-MeanIP computed from all dynamics; Method 5 – as Method 2 but registered to the computed 3D-MIP. Coronal MIP time series were then created for display, following the subtraction of PC1, with Matlab (MathWorks, R2020b, Massachusetts). A sixth subtracted data set was produced without registration.

The default affine registration parameters were employed, while for deformable registration the bending energy penalty weighting β was set = 10% and the grid spacing = 8 voxels.^12^

### Image reviewing

After processing, images were presented to three reviewers (a Consultant in Lymphovascular Medicine, Consultant Interventional Radiologist and a Medical Physicist, all with experience of lymphatic imaging) for quality ranking. Reviewers were requested to rank images from highest to lowest quality, which we considered would be less sensitive to inter-reader variability than if they provided subjective assessments with an overall quality score.

**Figure 1.**
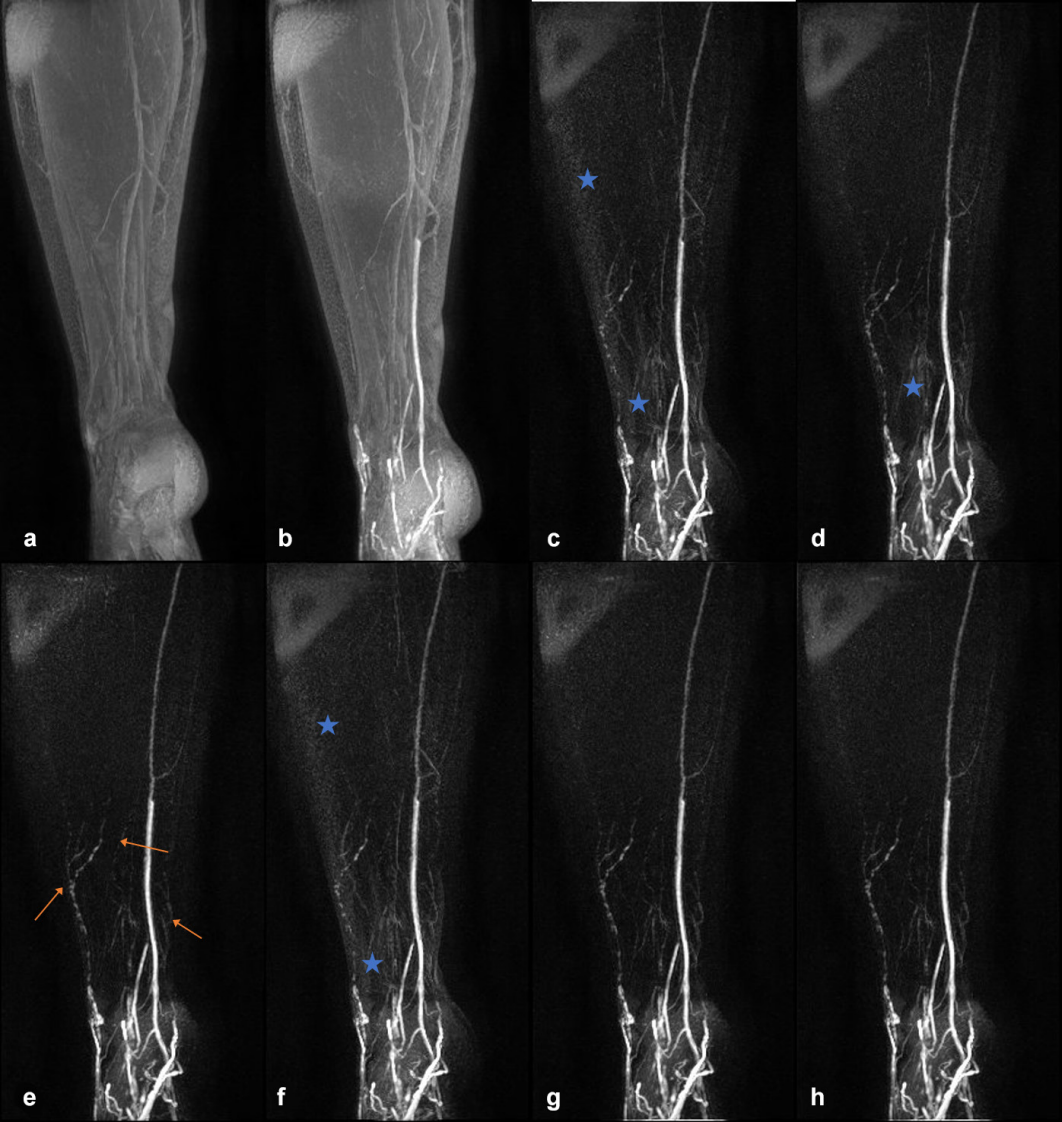
*T*_1_ weighted coronal MIP images from the right leg of an unaffected volunteer. a - pre-contrast MIP, b - post-contrast MIP, c - subtraction MIP produced without image registration, d - subtraction MIP produced after affine registration to PC1 (Method 1), e - subtraction MIP produced after affine and elastic registration to PC1 (Method 2), f - subtraction MIP produced after affine and elastic registration on a rolling basis (each volume registered to the preceding volume, Method 3), g - subtraction MIP produced after affine and elastic registration to the 3D mean intensity projection of the entire post-contrast series (Method 4), h - subtraction MIP produced after affine and elastic registration to the 3D maximum intensity projection of the entire post-contrast series (Method 5). Enhancement is observed in b, however additional vessel-like structures, assumed to be lymphatic based on their morphology, are visible in the subtraction images (orange arrows in e). Subjectively, less background signal and subtraction artefacts are apparent in e, g and h compared to c, d and f (see blue stars for examples of noisy or artefactual regions). All reviewers stated that subtraction images improved lymph vessel conspicuity here. b–h display data acquired approx. 20 minutes after contrast administration. c–h are displayed with the identical window/level. Average image ranks (**c–h**): 4.7, 5.0, 2.0, 5.3, 2.3, 1.3, where 1 indicates the best image quality. MIP, maximum intensity projection; PC1, first post-contrast volume.

Images were viewed with the freeware package MicroDicom. The original unregistered and unsubtracted coronal MIP series were also presented to reviewers for comparison. Six subtracted data sets were displayed for each participant (see Figure 1 for an example of the images displayed to the reviewers), with the reviewers blinded to their origin. The order the images were displayed in was randomised across data sets, and images were windowed on an individual basis.

For each of the five healthy individual’s limbs, the six subtracted series (one from each registration pipeline) were ranked in order from one (best) to six (worst) regarding the clarity of the image, degree of misregistration, and lymphatic anatomical detail. In addition, reviewers were asked if the subtraction data sets assisted in identifying lymphatic structures compared to the unsubtracted data. Intraclass correlation coefficient (ICC) was computed to assess inter-rater reliability of rankings and a Wilcoxon signed-rank non-parametric test performed to compare the rankings of all registration groups, pooled across reviewers, with the unregistered image rankings. All statistical analysis was performed with SPSS (IBM, SPSS Statistics 27.0, Chicago, IL).

### Signal enhancement characteristics

Signal enhancement curves were produced for two individuals: the affected limb of a participant diagnosed with unilateral lower limb lymphoedema in which little motion was visually detected, and the limb of the healthy volunteer in which unwanted motion was most evident. Signal was measured in a 3 × 3 × 3 voxel (2.1 × 2.1 × 2.1 mm) region of interest (ROI) of the unsubtracted data, after first identifying the vessel in the subtraction dynamic MIP image series. For efficiency, the ROI was positioned first on the unregistered image series and copied to the registered data sets. Due to displacements incurred by registration, small adjustments to the ROI position were required on occasion to ensure the ROI remained centred on the vessel. Average signal was measured over the first ~30 minutes of the DCE-MRL series using ImageJ v. 1.52 (National Institutes of Health, Maryland).

## Results

**Figure 3.**
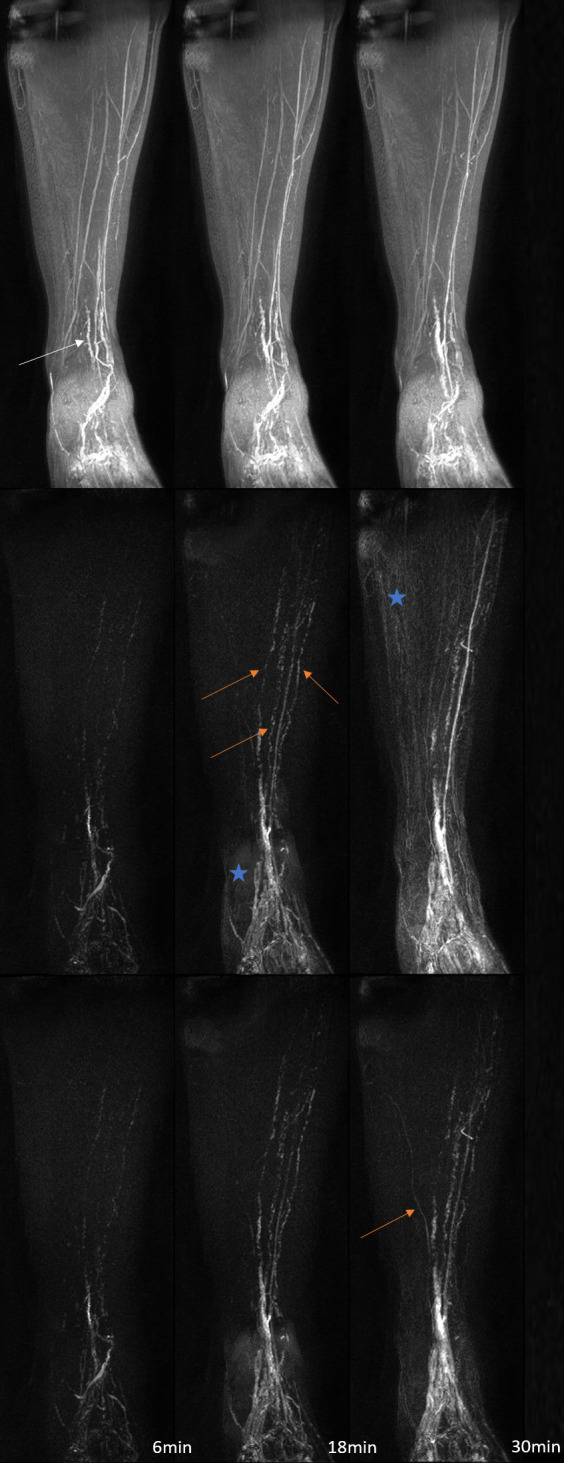
*T*_1_ weighted coronal MIP images from the right leg of a healthy volunteer shown at approximately 6, 18 and 30 minutes after the commencement of post-contrast imaging. The top row shows the unsubtracted coronal MIP images, while the middle and bottom rows show MIPs produced after subtraction of PC1 without prior registration (middle row) or after affine and elastic registration to the first post-contrast volume (bottom row). A vascular structure of presumed lymphatic origin can be seen in the unsubtracted images (white arrows), with additional structures appearing in subtraction data (orange arrows). Note how the background signal and subtraction artefacts (blue stars) increase with time in the subtracted but unregistered MIPs (middle row), while after registration this does not occur. Images in the middle and bottom row are displayed with the identical window/level. Average image ranks for the unregistered and images registered to PC1: 4.3 and 1.7 respectively. MIP, maximum intensity projection; PC1, first post-contrast volume.

**Figure 4.**
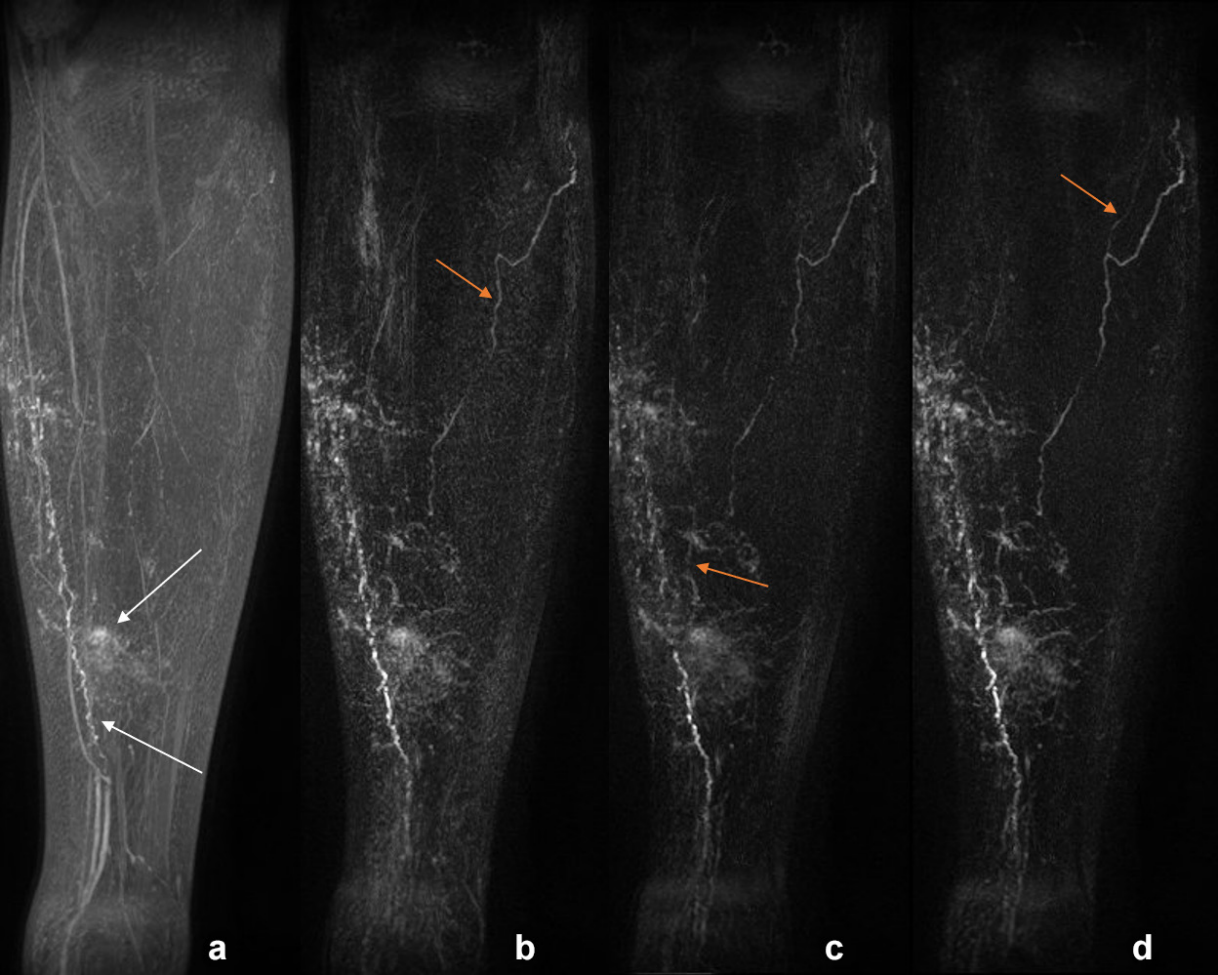
Left leg of a patient with unilateral primary lower limb lymphoedema, approx. 30 minutes post-contrast administration. A - coronal MIP, B - subtraction MIP produced without image registration, C - subtraction MIP produced after affine registration to PC1 (Method 1), D - subtraction MIP produced after affine and elastic registration to PC1 (Method 2). Tortuous lymphatics and contrast pooling in the skin can be seen in A (white arrow), with additional lymphatic vessels seen after subtraction (examples highlighted with orange arrows). Background signal is reduced after registration, particularly elastic (**D**). box inB–D are displayed with the identical window/level. MIP, maximum intensity projection; PC1, first post-contrast volume.

**Figure 2.**
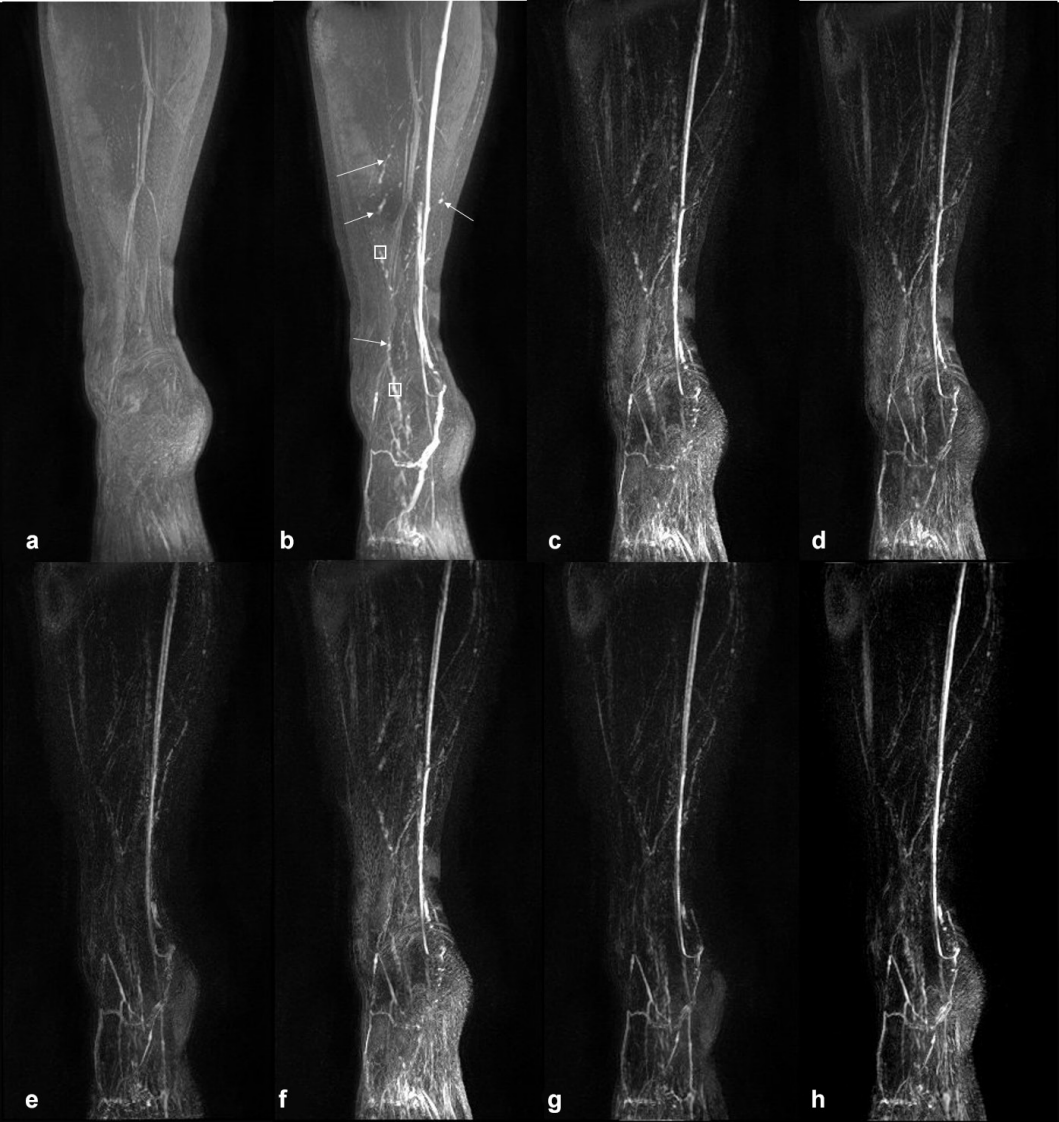
*T*_1_ weighted coronal MIP images from the right leg of the only unaffected volunteer for which two of the three reviewers stated that subtraction images did not improve lymphatic conspicuity. Multiple structures of presumed lymphatic origin are apparent in the unsubtracted images and were apparent in the first post-contrast MIP image (white arrows). a - pre-contrast MIP, b - post-contrast MIP, c - subtraction MIP produced without image registration, d - subtraction MIP produced after affine registration to PC1 (Method 1), e - subtraction MIP produced after affine and elastic registration to PC1 (Method 2), f - subtraction MIP produced after affine and elastic registration on a rolling basis (each volume registered to the preceding volume, Method 3), g - subtraction MIP produced after affine and elastic registration to the 3D mean intensity projection of the entire post-contrast series (Method 4), h - subtraction MIP produced after affine and elastic registration to the 3D maximum intensity projection of the entire post-contrast series (Method 5). The white boxes in b indicate the approximate locations of the regions of interest from which signal enhancement curves were produced (Figure 5) for this participant. C–H are displayed with the identical window/level. Average image ranks (**c–h**): 4.7, 3.7, 2.0, 5.3, 1.3, 3.7, where 1 indicates the best image quality. MIP, maximum intensity projection; PC1, first post-contrast volume.

DCE-MRL was technically successful in all participants, with the slight discomfort of injection well tolerated due to the use of topical anaesthesia. No adverse reactions were reported by any subject. Figures 1 and 2 demonstrate representative subtraction MIPs for two unaffected participants along with unregistered and unsubtracted MIP images pre- and post-contrast injection. Figure 3 shows a time course of subtraction images, in approximately 12 minute increments, for another healthy participant demonstrating MIP images produced with and without registration via Method 2. Note the increase in vessel-like structures present in images after registration and/or subtraction. Figure 4 shows subtraction MIPs of the leg of a participant diagnosed with unilateral primary lower limb lymphoedema prior and post registration via Method 1 or 2. An example series of registered and unregistered coronal MIP time series images are available in the supplementary data.

**Figure 5.**
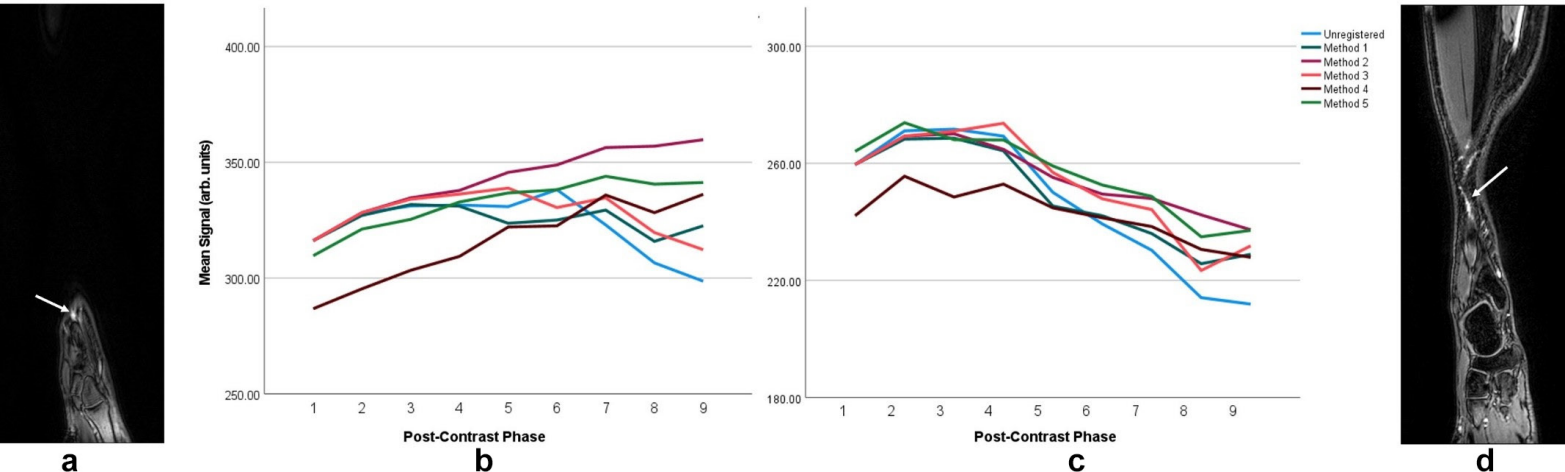
Signal enhancement characteristics in the limb of the unaffected individual who moved the most across the course of imaging (ankle moved substantially, the leg to a lesser extent). A and D show the central slice over which the signal was measured with arrows indicating the vessel interrogated (see Figure 2B for the locations on a coronal MIP image). These vessels are at the anterior aspect of the ankle and lower leg respectively, with the ankle vessel appearing to eventually drain to the vessel in the leg. In the ROI centred on the ankle, the signal in the images registered with different techniques show variable temporal behaviours with some showing consistent increase in signal (Methods 2, 4 and 5) and the remainder showing a signal decrease after an initial rise. Further up the limb, though the signal evolution varies somewhat between data sets, the overall pattern of signal change remains constant with a short initial increase followed by a gradual reduction. Method 1 - affine registration to the first post-contrast volume, Method 2 - elastic registration to PC1 following initial affine registration, Method 3 - affine and elastic registration on a rolling basis with each volume registered to the preceding, Method 4 - affine and elastic registration to themean intensity projection of the dynamic dataset, Method 5 - affine and elastic registration to the MIP of the dynamic data set. MIP, maximum intensity projection; PC1, first post-contrast volume.

Image subtraction, without prejudice to registration technique, was reported as improving vessel visibility in the majority of limbs; with reviewers stating subtraction improved vessel detail in 5/5, 4/5 and 4/5 cases respectively. The participant for which two reviewers reported no improvement is shown in Figure 2.

### Computation time

Implementing registration of dynamic data sets was a relatively lengthy process. A representative image data set (560 × 269 × 240 pixels, 16 post-contrast acquisitions) was registered using a system with 32 GB RAM and Intel^®^ Core^TM^ i7 3 GHz processor in: Method 1 = 63 minute, Method 2 = 211 minutes, Method 3 = 212 minutes, Method 4 = 204 minutes, and Method 5 = 201 minutes.

### Image ranking

The consistency between the three reviewers (inter-rater reliability) was assessed as moderate given the average ICC = 0.71 (95% confidence range = 0.47–0.85).

The results of image ranking are summarised in Table 1. Given the low number of cases and lack of normal distributions, data from all observers were pooled and Wilcoxon (non-parametric) signed-rank tests performed to compare ranks to the unregistered series. Significant differences (*p* < 0.05) in rankings were observed for four methods compared with the unregistered data. Methods 2, 4 and 5 had significantly lower ranks (*i.e.* higher quality) while Method 3 (rolling registration) was significantly higher ranked. Method 2 was rated as highest quality with an average rank ± standard deviation = 1.9 ± 0.9 (*p* = 0.006 when compared to unregistered images).

**Table 1.** Average reviewer rankings of the registration techniques (1 = best, 6 = worst) for *n* = 5 controls

	Unregistered	Method 1	Method 2	Method 3	Method 4	Method 5
**Reviewer 1**	4.0	4.0	1.8	4.8	2.8	2.0
**Reviewer 2**	3.2	4.8	2.6	5.0	1.6	2.2
**Reviewer 3**	4.6	4.0	1.4	5.2	2.4	3.2
**Mean ± Stdev**	3.9 ± 0.7	4.3 ± 0.5	1.9 ± 0.9	5.0 ± 0.2	2.3 ± 0.6	2.5 ± 0.6
***p* < 0.05?**	N/A	No	Yes	Yes^a^	Yes	Yes

The registration performed in each of the rightmost columns includes both an initial affine (Method 1) followed by an elastic registration, either to the first post-contrast phase (Method 2), on a rolling basis with each volume registered to the preceding (Method 3), to the mean intensity projection of the dynamic dataset (Method 4), or maximum intensity projection of the data set (Method 5). Method 1 includes only affine registration to the first post-contrast volume. Wilcoxon signed-rank tests showed significantly different rankings compared to those of the unregistered images for all but Method 1. For Method 3, this was a significantly worse ranking.

a Significantly worse image ranking compared to the unregistered data.

### Signal enhancement characteristics

In the unaffected individual with evident unwanted limb motion, lymphatic signal was seen to increase over the first ~15–20 minutes of imaging regardless of registration technique (Figure 5). The signal continued to rise for some registration techniques (Methods 2, 4, 5), consistent with contrast ‘wash-in’, and fell in all others.

**Figure 6.**
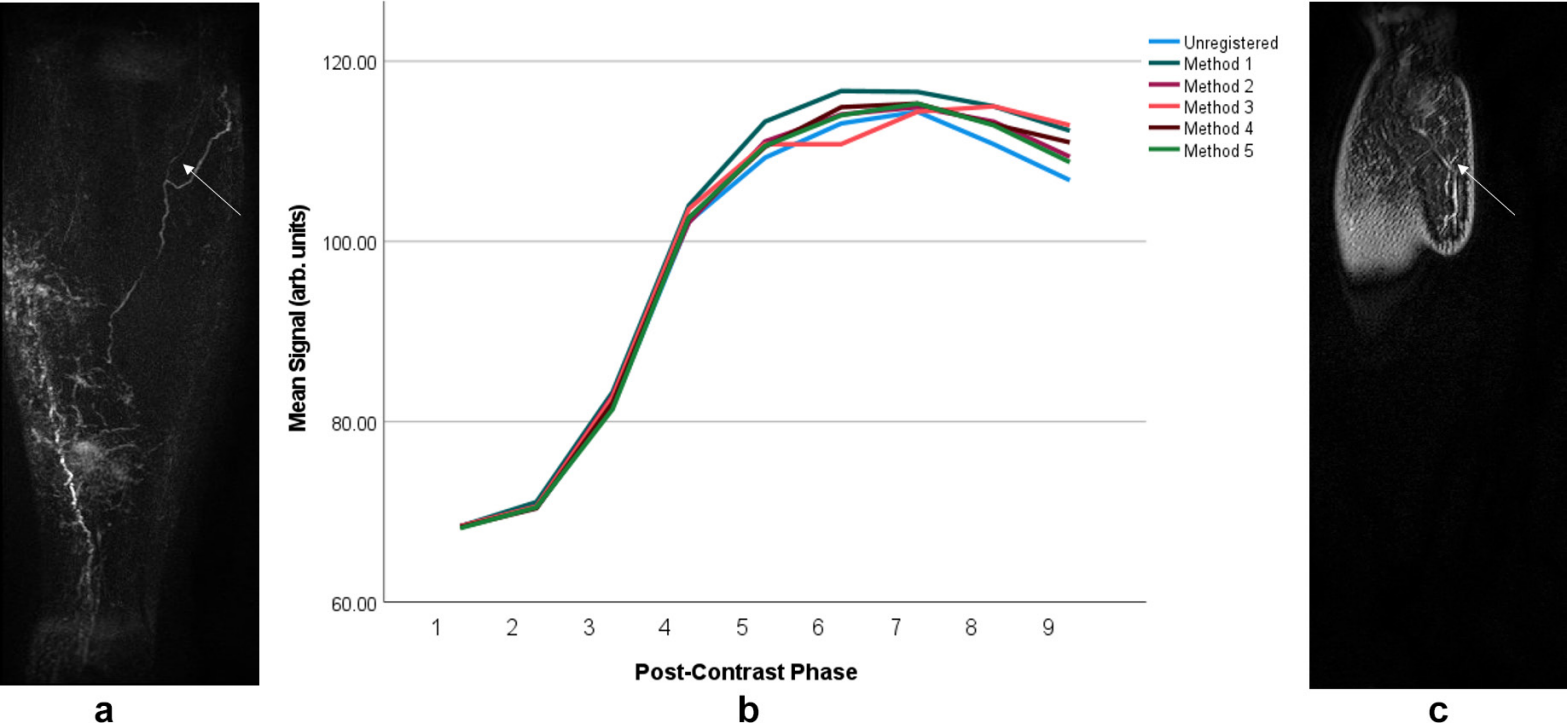
Signal enhancement curve measured in the limb of a unilateral lymphoedema patient. Frame a depicts the coronal maximum intensity projection image also shown in Figure 4D, while C shows the central slice over which the signal was measured. The vessel investigated was located in the posterior lateral aspect of the leg (see arrows). As seen in B, the signal in the images registered with different techniques show very similar temporal behaviour and magnitude. Visually there was very little motion across the dynamic series, hence this similarity. Method 1 - affine registration to PC1, Method 2 - elastic registration to PC1 following initial affine registration, Method 3 - affine and elastic registration on a rolling basis with each volume registered to the preceding, Method 4 - affine and elastic registration to the mean intensity projection of the dynamic dataset, Method 5 - affine and elastic registration to the maximum intensity projection of the dynamic data set. PC1, first post-contrast volume.

In the affected limb of a participant with unilateral primary lymphoedema, signal enhancement curves remained similar regardless of the registration technique as can be seen in Figure 6 (a signal enhancement curve obtained from the unaffected limb of the same participant is available in the supplementary data).

## Discussion

MRL provides both functional and anatomical lymphatic detail in disease states such as lymphoedema, however technical hurdles such as achieving optimal labelling of enhancing structures (*i.e.* differentiating lymphatic vessels from other enhancing features) and depiction of normal lymphatics remain. Our study demonstrates that registration and subtraction of DCE-MRL data sets can improve vessel visualisation in unaffected limbs. It also appears that the effects of limb motion and intensity variations are best accommodated with an elastic registration technique as the highest rated processing pipeline (Method 2) involved both affine and elastic registration.

Though the focus of our study was DCE-MRL in healthy volunteers, for which observing lymphatic vessels is reported as being more difficult than in individuals with lymphoedema, we also present images following registration and subtraction for a single lymphoedematous limb. While tortuous lymphatic vessels were clear in the maximum intensity projection images produced without further processing, additional lymphatic vessels became apparent following subtraction of the reference volume, with reduced background signals observed when the data were registered with an elastic approach (Figure 4). Studies focused on the use of these image processing techniques in lymphoedematous lower limbs are required to assess if this promising result is replicated in a larger patient cohort.

### Image ranking

As demonstrated in Figures 1, 3 and 4, without image registration anatomical features may be obscured in maximum intensity projected images following subtraction of a reference baseline image, while registration pipelines including elastic registration can reduce subtraction artefacts and background signal compared to affine registration alone. Registration pipelines employing a computed volume (Method 4: 3D-MeanIP and Method 5: 3D-MIP) were included in this study under the assumption that registration would be improved with a reference volume in which any lymphatic vascular enhancement would be evident. Images produced based on these two methods had significantly improved rankings compared to those without prior registration. However, images produced after registration with Method 2 consistently ranked as best quality, and so it appears that there is sufficient anatomical structure in the *T*_1_ weighted images (*e.g.* muscle, bone, facia) to facilitate reasonable registration regardless of the presence of vascular enhancing features.

It should be noted that these rankings were produced by reviewers asked to rank the image series based on image clarity, lymphatic anatomical detail and any apparent misregistration. Which of these parameters was most important to the review was not investigated however, and so it is possible that for each reviewer a different factor was most influential in their decision.

### Selection of reference baseline image

Throughout this study, registration to a post-contrast reference volume was performed. A pre-contrast reference was considered, however given the need for the participant to be moved through the scanner bore for contrast injection, which often necessitated coil repositioning (the coil tended to contact the bore during table movement and shift up the participants limbs), it was found that the imaged anatomy could vary between pre- and post-contrast phases. Studies investigating the use of both pre- and post-contrast reference volumes would allow the effects of this choice to be better understood.

The inability to use the pre-contrast volume as a baseline for subtraction here may explain why reviewers did not value subtraction images in the participant displayed in Figure 2 where clear enhancement was present in the first post-contrast image. Subtraction of PC1 from all subsequent images in this case will reduce the signal within enhanced vessels compared to the unsubtracted images and reduce their conspicuity rather than bringing them to the fore. Maximum intensity projection images may further compound this issue as, following subtraction, vessels with reducing signal (‘washing out’) compared to PC1 will be assigned negative signal values and lost in the MIP. A more appropriate receive coil selection, *e.g.* dedicated lower limb array, or use of the scanner body coil, as recently implemented,^17^ could alleviate this issue and provide even greater anatomical and enhancement time-course details. However, use of the native body coil will result in reduced signal-to-noise ratio and an inability to perform parallel imaging resulting in lower spatial or temporal resolution. Additionally, variability in uptake speed based on individual physiology and time between the initiation of contrast agent injection and subsequent imaging will also be important factors when the first post-contrast volume is used as the reference image. Though reducing the time between contrast injection and imaging may improve subtraction image quality, we recommend that investigators aim for consistency in contrast injection technique over reducing this delay. By employing a reduced injection rate, the pain associated with injection can be reduced, intradermal administration is promoted, which, when coupled with a consistent delay between injection and imaging, will improve reliability and tolerability of DCE-MRL studies.

### Signal enhancement characteristics

Signal enhancement curves were found to differ between registration methods when measured in a region of substantial motion: signal continued rising over the series when the images were registered with Methods 2, 4 and 5, those producing the highest quality images, however for the remainder of registration techniques, or lack thereof, signal peaked and began to reduce (Figure 5B). This suggests that prior registration can alter estimates of lymph dynamics when signal enhancement is used as a proxy for lymph flow. Higher up the limb, where motion was still present but less pronounced, the morphology of the signal curves remains consistent (slight uptake followed by signal reduction), however a more dramatic signal loss was observed in the unregistered data.

When little motion across the dynamic series was perceived, signal enhancement curves appeared similar regardless of registration technique, suggesting that registration may not be necessary for measuring signal changes in these cases (Figure 6). Nonetheless, we recommend the use of image registration as identifying enhancing structures was made easier by first visualising the vessel in the post-processed MIP image series. As can be seen in Figures 1, 3 and 4, subtly enhancing structures may only be apparent after registration pipelines including elastic registration are employed (excluding Method 3). This ability to visualise enhancing structures facilitates higher confidence in ROI placement and assessment of vessels, which could otherwise easily be missed or not identified as of a vascular origin.

## Conclusion

The lymphatic detail in DCE-MRL can be improved by employing image registration and subtraction of the first post-contrast dynamic volume in unaffected controls for which lymphatic vessel visualisation remains difficult. Encouraging results were also observed in a single patient with lymphoedma. The improved lymphatic detail in these images also facilitates the identification of regions in which to assess signal enhancement characteristics and potentially alters the measured signal time course when participant motion is present.

We believe that with an optimised protocol for the investigation of the lymphatics in healthy limbs our understanding and interpretation of the pathophysiology in lymphoedema will be improved. With optimal definition of lymphatic vessels, MRL is potentially the most promising investigation for imaging lymphatic vessels of both superficial and deep lymphatic systems. Future studies are required which investigate the use of registration techniques with different optimisation metrics, registration to the pre-contrast image (assuming a suitable coil is available), collection of venograms to mask out venous signal, and larger cohorts of participants including those affected by lymphoedema.

## Footnotes

* - note that both feet were injected with a Dotarem® containing solution, however many of the healthy individuals were injected with solutions containing different concentrations of Gadolinium in either foot to explore the effect on image quality.
